# A Phase-Field Study of Spinodal Decomposition Impeded by Irradiation in U-Mo and U-Mo-Zr Alloys

**DOI:** 10.3390/ma16247546

**Published:** 2023-12-07

**Authors:** Yong Lu, Xue Ni, Honghao Guo, Xiaoyi Huang, Dan Sun, Wenjie Li, Xingjun Liu, Cuiping Wang

**Affiliations:** 1 Fujian Key Laboratory of Surface and Interface Engineering for High Performance Materials, College of Materials, Xiamen University, Xiamen 361005, China; michellenix@163.com (X.N.); hhgsuper@163.com (H.G.); huangxiaoyi98@163.com (X.H.); lxj@xmu.edu.cn (X.L.); wangcp@xmu.edu.cn (C.W.); 2 Science and Technology on Reactor System Design Technology Laboratory, Nuclear Power Institute of China, Chengdu 610213, China; lwj04@163.com; 3 Department of Materials Science and Engineering, Harbin Institute of Technology, Shenzhen 518055, China

**Keywords:** phase-field method, U-Mo alloy, U-Mo-Zr alloy, spinodal decomposition, irradiation

## Abstract

The phase-field method, coupled with the micro-elastic model and irradiation-induced cascade mixing model, has been employed to investigate the spinodal decomposition in U-Mo and U-Mo-Zr alloys. The microstructure evolution of U-Mo or U-Mo-Zr alloys under different initial conditions, such as the alloy composition, aging temperature and irradiation intensity, were simulated to study the effect of cascade mixing on the miscibility gap, morphology and volume fraction of the decomposed phases. The simulation results demonstrate that irradiation-induced cascade mixing impedes the process of spinodal decomposition, and that irradiation shrinks the composition range of the miscibility gap in the alloys. Irradiation-induced cascade mixing slows down the anisotropic growth rate of the spinodal decomposition, yet this phenomenon can be weakened with increasing aging temperature. Adding an appropriate amount of Zr to a U-Mo alloy can effectively prevent the contraction of the miscibility gap caused by irradiation.

## 1. Introduction

With the wide application of nuclear energy, the safety and reliability of nuclear reactors have attracted more and more attention [1]. The irradiation-induced swelling behavior of metallic nuclear fuels could produce serious safety hazards under heat and irradiation conditions [2]. Uranium-based metallic nuclear fuels with γ-phases (Bcc structures) are considered as advanced nuclear fuels due to their advantages of thermal conductivity, irradiation performance and anti-swelling properties [3,4,5,6]. Recently, research on U-Zr alloys has indicated that the spinodal decomposition of the γ-phase can effectively inhibit irradiation swelling [7,8]. The γ-phase generally appears in uranium-based alloys, such as U-Mo, U-Nb, U-Zr and U-Mo-Zr alloys [9,10,11,12]. Therefore, the study of spinodal-decomposed microstructures with stable γ-phases in uranium-based metallic nuclear fuels has important guiding significance for the development of nuclear fuels.

Recently, because of their good irradiation stability, high-density fissionability, high thermal conductivity and low manufacturing cost [2,13], U-Mo and U-Mo-Zr alloys are considered as promising candidate fuels for fast breeder reactors. Various efforts have been made to study their mechanical properties [14,15], phase stability [4,16,17] and phase transformation [18,19]. The addition of Mo can effectively stabilize the γU-phase and improve the thermal conductivity and anti-swelling property [20], and the addition of Zr can effectively improve the stability of γU-phases over a wider temperature range [14]. It is reported that, with the microstructure of spinodal decomposition, the accumulation of fission gases and the swelling effect could be well detained in U-Mo alloys [21]. In addition, the results of Yao et al.’s research [7,8] showed that the spinodal decomposition formed nanoscale structures in U-Zr alloys after irradiation, and that this kind of microstructure could effectively prevent the irradiation swelling of fuel slugs. Our previous results [22] indicated that the effect of irradiation on microstructure evolution is opposite to that of spinodal decomposition; i.e., the solute atoms can be driven back into the solution phase during spinodal decomposition. However, the spinodal decomposition and de-mixing effect of U-Mo and U-Mo-Zr alloys under irradiation are still not clear.

The phase-field method is widely used and achieves successful applications in microstructure evolution under irradiation, such as phase separation [23,24,25,26,27], void formation and migration [28,29,30,31], gas bubble growth [32,33,34] and so on. The formation of various microstructures in Ag-Cu alloys associated with spinodal hardening under irradiation was predicted by Demange et al. [35] based on a phase-field model. Hu et al. [33,36] and Liang et al. [37] successfully developed phase-field models, and studied the formation mechanism of gas bubbles and the influence of bubble structures in U-Mo alloys under irradiation. The phase-field method coupled with the micro-elastic theory was employed by Yan et al. [26] to investigate the evolution of interstitial atoms and vacancies in Fe-Cr alloys under irradiation, which indicates that the phase-field micro-elastic theory can successfully describe the heterogeneity and spatial distribution of structures at the mesoscale [38,39]. The phase-field method is an intuitive way to understand microstructural evolution under irradiation in U-Mo and U-Mo-Zr alloys.

In the present study, we have employed the phase-field method coupled with a thermodynamic database to study the γ-phase’s spinodal decomposition in U-Mo and U-Mo-Zr alloys when exposed to radiation. The micro-elasticity model and cascade mixing model were used to reveal the cumulative effect of time-dependent local remixing and elastic-induced anisotropic growth preferences on microstructure evolution. The spinodal composition under different initial conditions (the alloy composition, aging temperature and irradiation intensity) in U-Mo and U-Mo-Zr alloys have been explored.

## 2. Model and Method

### 2.1. Phase-Field Model of U-Mo and U-Mo-Zr Alloy System

The phase-field method assumes that the interface between phases is diffusive; that is, the composition, structure and other properties of the region are continuously changing [40]. In this paper, the Cahn–Hilliard nonlinear evolution equation of concentration fields [41,42] is employed to investigate the evolution of spinodal decomposition in U-Mo and U-Mo-Zr alloys. Cahn and Hilliard hypothesized [43,44] that in an inhomogeneous system, the free energy in an infinitesimal volume depends on its composition and the composition of its surroundings, because different spatial configurations are not equal in energy for the same volume fraction. Considering the solid-state spinodal decomposition of the γ-phase, the total free energy (*F*) should include the chemical free energy, the interfacial gradient energy produced by uneven composition and elastic strain energy. The total free energy expression for the system can be written as follows:

(1) $$F=\int_{V}\left\{G_{chem}+\frac{1}{2}\kappa {\left(\nabla c\right)}^{2}\right\}dV+f_{el}$$

in which κ represents the gradient energy coefficient, *c* is the mole fraction of species Mo or Zr and $f_{el}$ denotes the elastic strain energy of the U-Mo or U-Mo-Zr alloy. The expression for the chemical free energy, *G_chem_*, of the γ-phase in U-Mo and U-Mo-Zr alloys can be defined as $G_{chem}=G^{m}/V_{m}$; here, *V_m_* symbolizes the molar volume and *G^m^* represents the molar Gibbs free energy. The molar Gibbs free energy in a U-Mo alloy can be expressed with the regular solution model:

(2) GA−Bm=∑i=A,BG0iγci+RT∑i=A,Bcilnci+cAcB∑j=0nLjA,Bγ(cA−cB)j

Here, *c_i_* is the atomic fraction of U and Mo. G0iγ represents the molar Gibbs free energy of pure U or Mo in the γ-phase, which is taken from Dinsdale’s SGTE database [45]. LjU,Moγ=aj+bjT is the interaction parameter between U and Mo, where *a_j_* and *b_j_* represent the optimized parameters.

In a U-Mo-Zr alloy, the molar Gibbs energy of the γ-phase can be expressed as follows:

(3) GA−B−Cm=∑i=A,B,CciG0i+RT∑i=A,B,Ccilnci+GEγGEγ=cAcBLA,Bγ+cAcCLA,Cγ+cBcCLB,Cγ+cAcBcCLA,B,Cγ

where *c_i_* is the atomic fraction of U, Mo and Zr. G0iγ represents the molar Gibbs free energy of pure U, Mo and Zr. GEγ is the excess free energy corresponding to the heat of mixing. The thermodynamic parameters of the U-Mo and U-Mo-Zr system utilized in this work are imported from with the CALPHAD database [12,46].

The phase-field method is a computational method used to simulate the dynamics of temporal microstructures. It involves analyzing non-linear evolution equations with both non-conserved and conserved order parameters to study their evolution over time. The compositional evolution in the γ-phase of the U-Mo alloy, which leads to spinodal decomposition, can be explained using the Cahn–Hilliard diffusion equation [47]:

(4) $$\frac{\partial c}{\partial t}=\nabla \cdot \left[M\nabla \left(\frac{\partial G_{chem}}{\partial c}-\kappa \nabla^{2}c+\frac{\delta f_{el}}{\delta c}\right)\right]+{\frac{\partial c}{\partial t}|}_{mixing}$$

in which *c* represents the nominal composition of Mo and *t* is time. The term outside the parentheses in the equation refers to the cascade mixing brought on by cascade damage, while the term inside the parentheses represents thermal diffusion driven by the gradient of diffusion potential on the right side of the equal sign. *M* represents the chemical mobility and it can be described using Darken’s equation [48]:

(5) $$M=c\left(1-c\right)\left[cM_{U}+\left(1-c\right)M_{Mo}\right]$$

Here, *M_U_* and *M_Mo_* represent the atomic mobilities of U and Mo, respectively. These mobilities are connected to the atomic diffusion coefficient, *D_i_*, which is defined in Einstein’s relation, $M_{i}=D_{i}/RT\left(i=U{,}_{}Mo\right)$, where T denotes the absolute temperature, and R represents the gas constant.

In the U-Mo-Zr alloy, the phase decomposition of the ternary system is also controlled based on the ternary Cahn–Hilliard diffusion equation [49]:

(6) $$\begin{array}{l} \frac{\partial c_{B}}{\partial t}=\nabla \cdot \left[\begin{array}{l} L_{BB}\nabla \left(\frac{\partial G_{chem}}{\partial c_{B}}+2\kappa_{BB}\nabla^{2}c_{B}-2\kappa_{BC}\nabla^{2}c_{C}+\frac{\delta f_{el}}{\delta c_{B}}\right)\\ +L_{BC}\nabla \left(\frac{\partial G_{chem}}{\partial c_{C}}+2\kappa_{BC}\nabla^{2}c_{B}-2\kappa_{CC}\nabla^{2}c_{C}+\frac{\delta f_{el}}{\delta c_{C}}\right) \end{array}\right]+{\frac{\partial c_{B}}{\partial t}|}_{mixing}\\ \frac{\partial c_{C}}{\partial t}=\nabla \cdot \left[\begin{array}{l} L_{CB}\nabla \left(\frac{\partial G_{chem}}{\partial c_{B}}+2\kappa_{BB}\nabla^{2}c_{B}-2\kappa_{BC}\nabla^{2}c_{C}+\frac{\delta f_{el}}{\delta c_{B}}\right)\\ +L_{CC}\nabla \left(\frac{\partial G_{chem}}{\partial c_{C}}+2\kappa_{BC}\nabla^{2}c_{B}-2\kappa_{CC}\nabla^{2}c_{C}+\frac{\delta f_{el}}{\delta c_{C}}\right) \end{array}\right]+{\frac{\partial c_{C}}{\partial t}|}_{mixing} \end{array}$$

where *c_i_* represents the local composition of element *i*. The subscript numbers *i* = *A*, *B*, *C* denote U, Mo and Zr, respectively. The gradient energy coefficients are $\kappa_{BB}=\kappa_{A}+\kappa_{B}$, $\kappa_{BC}=\kappa_{CB}=\kappa_{A}$ and $\kappa_{CC}=\kappa_{A}+\kappa_{C}$. $\frac{\delta f_{el}}{\delta c_{B}}$ and $\frac{\delta f_{el}}{\delta c_{C}}$ are variational derivatives of elastic energy. The Onsager coefficients (*L_ij_*) are determined by the intrinsic diffusion coefficient (*D_i_*) and the expression is defined in Equation (7), in which the intrinsic diffusion coefficient (*D_i_*) used in the simulation is shown in Table 1 [50,51].

(7) $$\begin{array}{l} L_{BB}=\frac{c_{B}}{RT}\cdot [c_{A}c_{B}D_{A}+{\left(1-c_{B}\right)}^{2}D_{B}+c_{B}c_{C}D_{C}]\\ L_{CC}=\frac{c_{C}}{RT}\cdot [c_{A}c_{C}D_{A}+c_{B}c_{C}D_{B}+{\left(1-c_{C}\right)}^{2}D_{C}]\\ L_{BC}=L_{CB}=\frac{c_{B}c_{C}}{RT}\cdot [c_{A}D_{A}-(1-c_{B})D_{B}-(1-c_{C})D_{C}] \end{array}$$

To deal with cascade mixing, the simulation makes use of the continuum atom relocation model created by Enrique and Bellon [52]. In this model, a distribution function with an average relocation distance (*R_L_*) can approximate the mixing range well, which is a magnitude close to the nearest-neighbor distance [53]. The forced cascade mixing leads to the change in local composition, given as follows [52,54]:

(8) $${\frac{\partial c}{\partial t}|}_{mixing}=-\Gamma \left(c-{\langle c\rangle }_{R_{L}}\right)$$

where Γ represents the atomic relocation frequency, which is correlated with the irradiation displacement rate, and it can be defined as Γ=λ⋅bΦ, in which *b* represents the parameter that characterizes the number of permutations for each atomic displacement, and Φ represents the rate of irradiation displacement. According to L’vov et al.’s result [55], the parameter *λ* can be shown as $\lambda =\frac{f_{v}c_{v}D_{v}+f_{i}c_{i}D_{i}}{fc_{v}D_{v}}=1+\frac{f_{i}\left(c_{i}D_{i}-c_{v}D_{v}\right)}{fc_{v}D_{v}}$, where the diffusion coefficient of vacancy is *D_v_ =* 10^−16^ m^2^ s^−1^, the interstitial diffusion coefficient is *D_i_ =* 10^−10^ m^2^ s^−1^ and the range of vacancy and interstitial concentration are *c_v_* = 10^−8^~10^−4^ and *c_i_* = 10^−14^~10^−10^. The dimensionless interstitial and vacancy correlation factors can be represented as *f_v_* and *f_i_*, respectively. Therefore, the parameter *λ* is treated as a constant with a value of 1, and the reorientation frequency is considered to be approximately equal to the irradiation displacement, which is defined as Γ ≈ *b*Φ. And ${\langle c\rangle }_{R_{L}}$ is the finite average of the concentration profile around a given spatial point, weighted using a normalized function [56,57,58]:

(9) $${\langle c\rangle }_{R_{L}}={\int \omega_{R_{L}}\left(r-\;r^{\prime }\right)}_{}c\left(r^{\prime }\right)d\;r^{\prime }$$

$\omega_{R_{L}}$ is the normalized distribution of atomic relocations, *R_L_*, which can be described using a Gaussian distribution as follows [54]:

(10) $$\omega_{R_{L}}\left(r-\;r^{\prime }\right)={\left(\frac{3}{2\pi R_{L}{}^{2}}\right)}^{\frac{3}{2}}\exp\left(-\frac{3{\left|r-\;r^{\prime }\right|}^{2}}{2R_{L}{}^{2}}\right)$$

where, **r** corresponds to the spatial location. Both *R_L_* and *b* depend on the properties of the cascades, and therefore, on the type of high-energy particles. In the Bcc lattice, the value of *R_L_* is usually approximately the nearest interatomic distance [59]. It is anticipated that there will be more substitutions than the model’s forecast value provided by Norgett et al. [60], taking into account that atomic displacements of Frenkel pairs are not generated during the cascade cooling process. The value range of *b* is chosen as ~1 for electrons while ranging from approximately 30 to 100 for neutrons and heavy ions [58]. Because the effect of the change in dose rate is less than one order of magnitude, the results of the present research are not particularly sensitive to a particular value of *b*. Therefore, the values of *R_L_* and *b* used in the simulation for neutron/heavy-ion irradiations are shown in Table 1.

### 2.2. Elastic Energy Model

Elastic energy mainly comes from three factors: (1) the degree of lattice mismatch between phases; (2) the inhomogeneity of the elastic constant; and (3) the external stress applied. This paper focuses on the elastic energy generated by the lattice mismatch between γ1 and γ2 phases of U-Mo and U-Mo-Zr alloys, which is also the main source of elastic energy. Therefore, it is assumed that the elastic constants of the γ1 phase and γ2 phase are the same, and there is no external stress in the alloy system studied.

According to Khachaturyan’s micro-elasticity theory, the elastic strain in the simulation brought on by the inhomogeneity of lattice mismatching in the alloys can be described as follows [38]:

(11) $$\varepsilon_{ij}^{el}=\varepsilon_{ij}^{}-\varepsilon_{ij}^{0}$$

where $\varepsilon_{ij}$ is the internal strain, which denotes the total strain’s assumed linear elasticity and is measured with the regard to a reference lattice. And $\varepsilon_{ij}^{0}=\delta_{ij}\varepsilon_{0}$ is the stress-free strain; here, $\delta_{ij}$ represents the Kronecker delta function [61]. $\varepsilon_{0}=1/a_{0}\left(da/dc\right)$ is the constituent expansion coefficient of the lattice parameter, where *a*_0_ and *a* represent the average lattice constant of the initial concentration (*c_0_*) and concentration (*c*) in a U-Mo alloy. The stress-free strain, $\varepsilon_{ij}^{0}$, in the U-Mo alloy is chosen as 0.0516. In the model, it is considered that the alloy system is elastically uniform and the local displacement field is periodic. Consequently, the elastic strain energy in reciprocal space can be expressed by employing Khachaturyan’s micro-elastic theory [38]:

(12) $$f_{el}=\frac{1}{2}\int \left\{\frac{d^{3}k}{{\left(2\pi \right)}^{3}}\left[B\left(n\right){\left|\tilde{c}\left(k\right)\right|}^{2}\right]\right\}$$

in which k is the Fourier wave vector, $n=\frac{k}{\left|k\right|}$ represents the unit vector in reciprocal space and $\tilde{c}\left(k\right)$ is the Fourier transform of composition *c*. The elastic interaction energy is $B\left(n\right)=\lambda_{ijkl}\varepsilon_{ij}^{0}\varepsilon_{kl}^{0}-n_{i}\sigma_{ij}^{0}\Omega_{jk}\left(n\right)\sigma_{kl}^{0}n_{l}$, where $\lambda_{ijkl}$ represents the elastic modulus tensor. According to the Hook’s law, $\sigma_{ij}=C_{ijkl}\varepsilon_{kl}^{el}$ is the stress and $\Omega_{jk}\left(n\right)=\lambda_{ijkl}n_{i}n_{l}$ is the inverse Green tensor.

Regarding a ternary substitutional alloy that comprises A, B and C atomic species, the stress-free strain, $\varepsilon_{ij}^{0}$, is given as $\varepsilon_{ij}^{0}=\sum_{p=0}^{1}\varepsilon_{ij}^{\left(p\right)}\theta_{p}$, where $\theta_{0}=c_{B}-c_{B}^{0}$, $\theta_{1}=c_{c}-c_{c}^{0}$, and $\varepsilon_{ij}^{\left(p\right)}$ is the position-independent portion of the stress-free strain field connected to $\theta_{p}$ [62]. The stress-free strains in the ternary alloy system are assume to be dilatational: $\varepsilon_{ij}^{\left(0\right)}=\varepsilon_{\alpha \beta }\delta_{ij}$ and $\varepsilon_{ij}^{\left(1\right)}=\varepsilon_{\alpha \gamma }\delta_{ij}$, where $\varepsilon_{\alpha \beta }$ and $\varepsilon_{\alpha \gamma }$ denote the lattice expansion coefficients of *c_B_* and *c_C_*, respectively. $\varepsilon_{\beta \gamma }=\varepsilon_{\alpha \beta }-\varepsilon_{\alpha \gamma }$ denotes the difference between $\varepsilon_{\alpha \beta }$ and $\varepsilon_{\alpha \gamma }$. The stress-free strain, $\varepsilon_{ij}^{\left(0\right)}$ and $\varepsilon_{ij}^{\left(1\right)}$, in the U-Mo-Zr alloy is chosen as 0.050 and 0.038, respectively. Therefore, the elastic strain energy in the reciprocal space is given as follows [63]:

(13) $$f_{el}=\frac{1}{2}\sum_{p,q=0}^{1}\int \left[\frac{d^{3}k}{{\left(2\pi \right)}^{3}}B_{pq}\left(n\right){\tilde{\theta }}_{p}\left(k\right){\tilde{\theta }}_{q}^{*}\left(k\right)\right]$$

Here, ${\tilde{\theta }}_{p}\left(k\right)$ represents the Fourier transform of $\theta_{p}\left(r\right)$, and the elastic interaction energy between *θ_p_* and *θ_q_* is $B_{pq}\left(n\right)=\lambda_{ijkl}\varepsilon_{ij}^{\left(p\right)}\varepsilon_{kl}^{\left(q\right)}-n_{i}{\hat{\sigma }}_{ij}^{\left(p\right)}\Omega_{jk}\left(n\right){\hat{\sigma }}_{kl}^{\left(q\right)}n_{l}$. ${\tilde{\theta }}_{}^{*}$ is the complex conjugate of $\tilde{\theta }$. The variational derivatives of the elastic energy with regard to the compound field variables in reciprocal space are derived based on the expression of elastic energy in Fourier space (Equation (13)) [63], and the change in the functional relative to the function is considered:

(14) $$\begin{array}{l} {\left[\frac{\delta f_{el}}{\delta c_{B}}\right]}_{k}={\left[\frac{\delta f_{el}}{\delta \theta_{0}}\right]}_{k}=B_{00}{\tilde{\theta }}_{0}+B_{01}{\tilde{\theta }}_{1}\\ {\left[\frac{\delta f_{el}}{\delta c_{C}}\right]}_{k}={\left[\frac{\delta f_{el}}{\delta \theta_{1}}\right]}_{k}=B_{01}{\tilde{\theta }}_{0}+B_{11}{\tilde{\theta }}_{1} \end{array}$$

Here, the symbol ${\left[\right]}_{k}$ shows that the quantity within the brackets is Fourier-transformed.

### 2.3. Numerical Methods

In our simulation, the composition evolution equation can be substituted from Equation (8) into Equation (4) or Equation (6). For the convenience of numerical calculation, the composition evolution equation in the binary U-Mo system can be transformed into a dimensionless form, and the expression is shown as follows:

(15) $$\frac{\partial c^{*}}{\partial t^{*}}=\nabla^{*}\cdot \left[M^{*}\nabla^{*}\left(\frac{\partial G_{chem}^{*}}{\partial c^{*}}-\kappa^{*}{\left(\nabla^{*}\right)}^{2}c^{*}+\frac{\delta f_{el}^{*}}{\delta c^{*}}\right)\right]-\Gamma^{*}\left(c^{*}-{\langle c^{*}\rangle }_{R_{L}{}^{*}}\right)$$

The dimensionless form of the composition evolution equation in the ternary U-Mo-Zr system is shown as follows:

(16) $$\begin{array}{l} \frac{\partial c_{B}^{*}}{\partial t^{*}}=\nabla^{*}\cdot \left[\begin{array}{l} L_{BB}^{*}\nabla^{*}\left(\frac{\partial G_{chem}^{*}}{\partial c_{B}^{*}}+2\kappa_{BB}^{*}{\left(\nabla^{*}\right)}^{2}c_{B}^{*}-2\kappa_{BC}^{*}{\left(\nabla^{*}\right)}^{2}c_{C}^{*}+\frac{\delta f_{el}^{*}}{\delta c_{B}^{*}}\right)\\ +L_{BC}^{*}\nabla^{*}\left(\frac{\partial G_{chem}^{*}}{\partial c_{C}^{*}}+2\kappa_{BC}^{*}{\left(\nabla^{*}\right)}^{2}c_{B}^{*}-2\kappa_{CC}^{*}{\left(\nabla^{*}\right)}^{2}c_{C}^{*}+\frac{\delta f_{el}^{*}}{\delta c_{C}^{*}}\right) \end{array}\right]-\Gamma^{*}\left(c_{B}^{*}-{\langle c_{B}^{*}\rangle }_{R_{L}{}^{*}}\right)\\ \frac{\partial c_{C}^{*}}{\partial t^{*}}=\nabla^{*}\cdot \left[\begin{array}{l} L_{CB}^{*}\nabla^{*}\left(\frac{\partial G_{chem}^{*}}{\partial c_{B}^{*}}+2\kappa_{BB}^{*}{\left(\nabla^{*}\right)}^{2}c_{B}^{*}-2\kappa_{BC}^{*}{\left(\nabla^{*}\right)}^{2}c_{C}^{*}+\frac{\delta f_{el}^{*}}{\delta c_{B}^{*}}\right)\\ +L_{CC}^{*}\nabla^{*}\left(\frac{\partial G_{chem}^{*}}{\partial c_{C}^{*}}+2\kappa_{BC}^{*}{\left(\nabla^{*}\right)}^{2}c_{B}^{*}-2\kappa_{CC}^{*}{\left(\nabla^{*}\right)}^{2}c_{C}^{*}+\frac{\delta f_{el}^{*}}{\delta c_{C}^{*}}\right) \end{array}\right]-\Gamma^{*}\left(c_{C}^{*}-{\langle c_{C}^{*}\rangle }_{R_{L}{}^{*}}\right) \end{array}$$

where $t^{*}=\frac{t\tilde{D}}{l^{2}}$, $\nabla^{*}=l\nabla$, $M^{*}=\frac{RTM}{\tilde{D}}$, $L^{*}=\frac{RTL}{\tilde{D}}$, $G_{chem}^{*}=\frac{V_{m}G_{chem}}{RT}$, $\kappa^{*}=\frac{\kappa }{RTl^{2}}$, $f_{el}^{*}=\frac{V_{m}f_{el}}{RT}$, $\Gamma^{*}=\frac{\Gamma l^{2}}{\tilde{D}}$, $R_{L}{}^{*}=\frac{R_{L}}{l}$ and $V_{m}=\frac{N_{A}a^{3}}{2}$. Here, l represents the grid length and is chosen based on the average lattice parameters of U and Mo for *c* = 1/2, or U, Mo and Zr for *c* = 1/3. The dimensionless grid size in the simulation is $\Delta x^{*}=\Delta y^{*}=1.0$. $\tilde{D}=c_{U}D_{U}+c_{Mo}D_{Mo}+c_{Zr}D_{Zr}$ is the inter-diffusion coefficient of the U-Mo or U-Mo-Zr alloy, and the value can be calculated from the temperature (*T*), the concentration of Mo or Zr and the intrinsic diffusion coefficients from Table 1. The size of the simulation cell is $256\Delta x^{*}\times 256\Delta y^{*}$, *N_A_* is the Avogadro constant and $\Delta t^{*}$ is the dimensionless time step. The anisotropy elastic constants ($C_{ij}$) of γ-U, γ-Mo, β-Zr and the lattice parameters, $a_{i}\left(i=U,Mo\;or\;Zr\right)$, are shown in Table 1 [64,65,66,67,68]. In the simulated region, the crystallographic orientation of the vertical direction is [01], and the horizontal direction is [10].

**Table 1:** Summary of the parameters used in the simulation.

Parameter	Value	Reference
Gradient energy coefficient (mol·m^2^·J^−1^)	$\kappa =5.7\times 10^{-15}$ (In U-Mo) $\kappa =4.2\times 10^{-15}$ (In U-Mo-Zr)	[69]
Gas constant (J·mol^−1^·K^−1^)	R=8.314	This work
Grid length (m)	$l=3.289\times 10^{-10}$ (in U-Mo) $l=3.386\times 10^{-10}$ (in U-Mo-Zr)	[64,66,67]
Intrinsic diffusion coefficient of U (m^2^·s^−1^)	$D_{U}=6.4\times 10^{-6}\exp\left(\frac{-165900_{}\;J\cdot mol^{-1}}{RT}\right)$	[50]
Intrinsic diffusion coefficient of Mo (m^2^·s^−1^)	$D_{Mo}=7.8\times 10^{-6}\exp\left(\frac{-185200_{}\;J\cdot mol^{-1}}{RT}\right)$	[50]
Intrinsic diffusion coefficient of Zr (m^2^·s^−1^)	$D_{Zr}=5.1\times 10^{-13}\exp\left(\frac{-51000_{}\;J\cdot mol^{-1}}{RT}\right)$	[51]
Lattice constant of γ-U (m)	$a_{U}=3.427\times 10^{-10}$	[66]
Molar volume of γ-U (m^3^·mol^−1^)	$V_{m}^{U}=1.212\times 10^{-5}$	[66]
Lattice constant of γ-Mo (m)	$a_{Mo}=3.150\times 10^{-10}$	[64]
Molar volume of γ-Mo (m^3^·mol^−1^)	$V_{m}^{Mo}=9.408\times 10^{-6}$	[64]
Lattice constant of β-Zr (m)	$a_{Zr}=3.582\times 10^{-10}$	[67]
Molar volume of β-Zr (m^3^·mol^−1^)	$V_{m}^{Zr}=1.383\times 10^{-5}$	[67]
Elastic constants of γ-U (GPa)	$C_{11}=86,C_{12}=155,C_{44}=37$	[66]
Elastic constants of γ-Mo (GPa)	$C_{11}=466,C_{12}=157,C_{44}=103$	[68]
Elastic constants of β-Zr (GPa)	$C_{11}=87,C_{12}=92,C_{44}=27$	[65]
Replacements per displacement	b=50	[58,70]
Average relocation distance (m)	$R_{L}=3.289\times 10^{-10}$ (in U-Mo) $R_{L}=3.386\times 10^{-10}$ (in U-Mo-Zr)	[64,66,67]
Time steps (s)	$\Delta t^{*}=5\times 10^{-3}$	This work

## 3. Results

### 3.1. Simulation of Spinodal Decomposition in U-Mo and U-Mo-Zr Alloys

**Figure 1:**
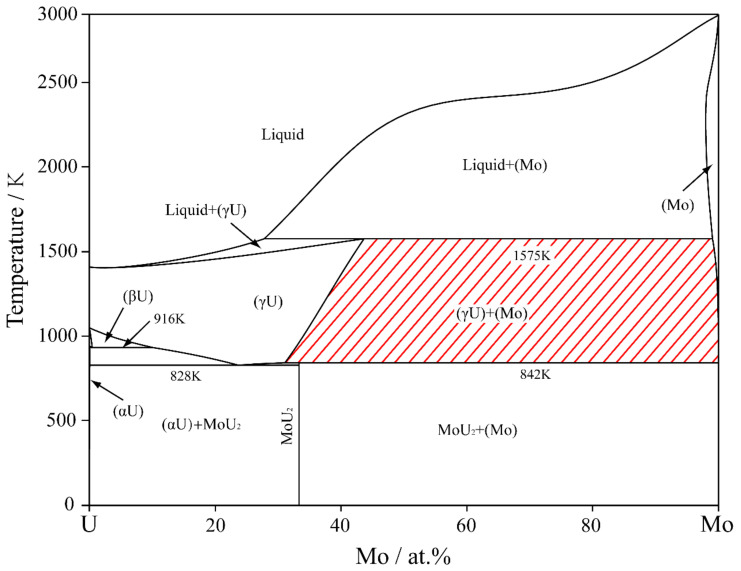
Calculated U-Mo equilibrium phase diagram [9].

**Figure 2:**
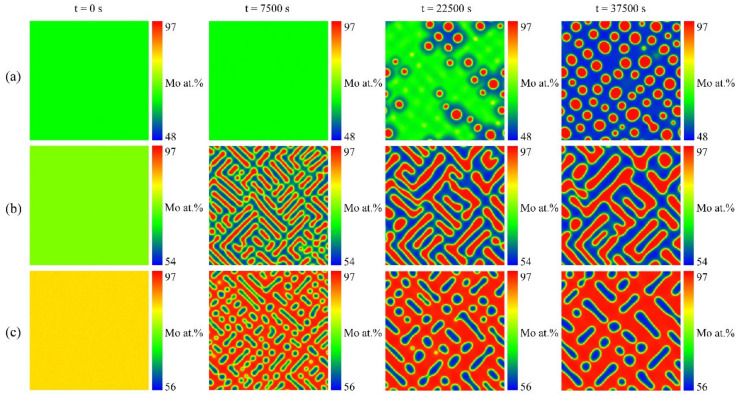
Simulation results of the spinodal decomposition under different initial alloy compositions of U-Mo alloy at 1273 K. Rows (**a**–**c**) correspond to U-65 at.% Mo, U-75 at.% Mo and U-85 at.% Mo alloys, respectively.

For the U-Mo binary alloy, there is a wide miscibility gap in the temperature range of 842 K to 1575 K, and the calculated phase diagram can be seen in Figure 1 [9]. Simulations were performed to study the spinodal decomposition in U-Mo alloys with various initial compositions, taking into account the elastic misfit between the two phases. Figure 2 illustrates the microstructure evolution of the γ-phase during spinodal decomposition in U-65 at.% Mo, U-75 at.% Mo, and U-85 at.% Mo alloys aged at 1273 K for 0, 7500, 22,500 and 37,500 s, in which the Mo-enriched γ-phase and U-enriched γ-phase are presented in dark red and dark blue, respectively. The color bars represent the Mo concentration profiles. For the U-65 at.% Mo alloy, the embedding of the Mo-enriched γ-phase into the U-enriched γ-phase matrix results in the formation of a round pattern. It can be seen that the Mo-enriched γ-phase in the U-75 at.% Mo alloy is in irregular and interconnected plane-like or maze-like shapes during spinodal decomposition, while the U-enriched γ-phase is embedded into the Mo-enriched γ-phase matrix in the U-85 at.% Mo alloy and forms an ellipsoidal shape. The rate of spinodal decomposition and volume fraction of the Mo-enriched γ-phase increase with higher concentrations of Mo in the U-Mo alloy.

**Figure 3:**
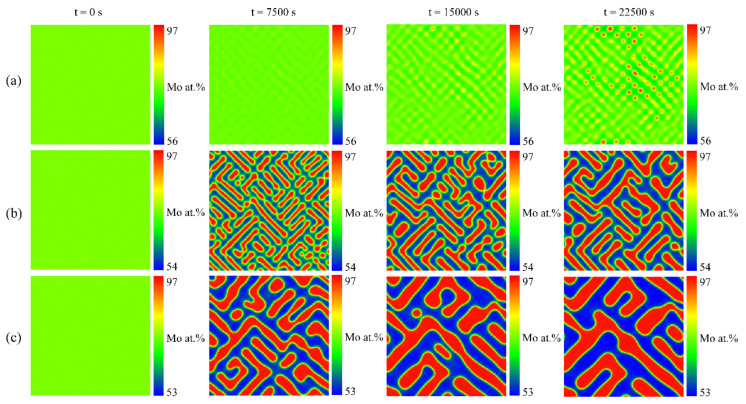
Simulation results of the spinodal decomposition in U-75 at.% Mo alloy at different temperatures. Rows (**a**–**c**) correspond to the temperatures at 923 K, 1273 K and 1473 K, respectively.

**Figure 4:**
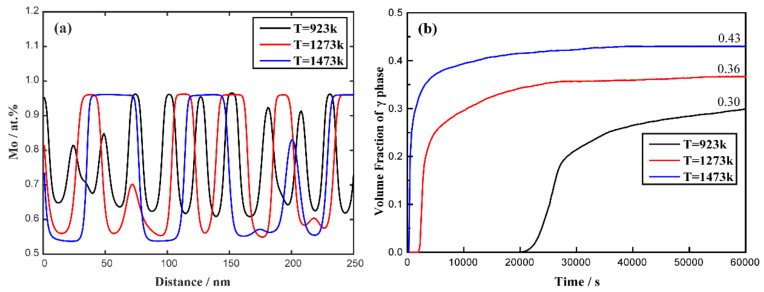
Simulation results of the U-75 at.% Mo alloy at different temperature (923 K, 1273 K and 1473 K): (**a**) the concentration profiles at the time of 22,500 s; (**b**) the volume fraction as a function of time.

The microstructure evolutions of the U-75 at.% Mo alloy at 923, 1273 and 1473 K are shown in Figure 3, which reflects the effects of different temperatures on spinodal decomposition. It can be seen that the morphology evolution rate of the M-enriched γ-phase accelerates with the increase in temperature. The size of the Mo-enriched γ-phase increases while the number of particles decreases, and the morphology of the Mo-enriched γ-phase becomes plane-like. Figure 4a shows the concentration profiles of the Mo-enriched γ-phase in the U-75 at.% Mo alloy under different temperatures at the time of 22,500 s. Upon temperature elevation, the fluctuation range of the composition gradually increases, and the Mo-enriched γ-phase reaches an equilibrium composition earlier. Figure 4b depicts the volume fraction evolution of the Mo-enriched γ-phase in the U-75 at.% Mo alloy at different temperatures. It is evident that the volume fraction of the Mo-enriched γ-phase increases significantly at high temperatures. Specifically, at 60,000 s, the equilibrium volume fractions of the Mo-enriched γ-phase are 30%, 36% and 43% for temperatures of 923 K, 1273 K and 1473 K, respectively.

**Figure 5:**
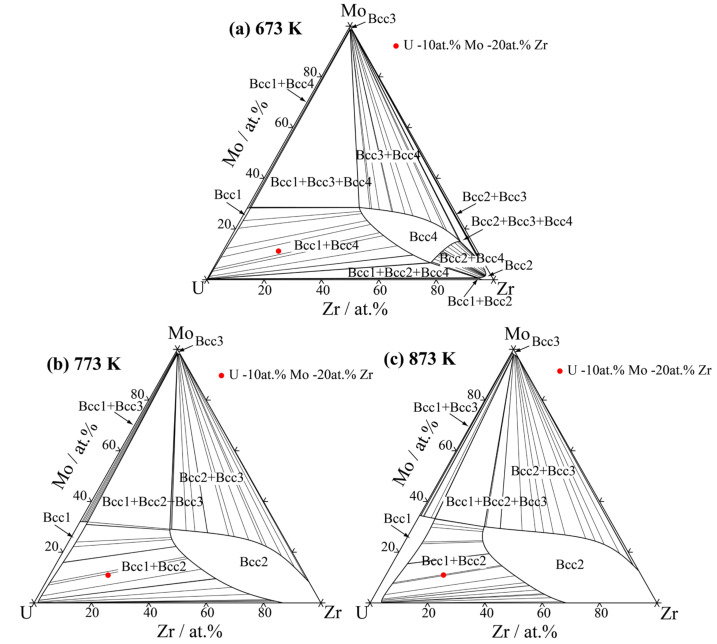
The calculated isothermal sections of U-Mo-Zr alloy: (**a**) 673 K, (**b**) 773 K, (**c**) 873 K.

**Figure 6:**
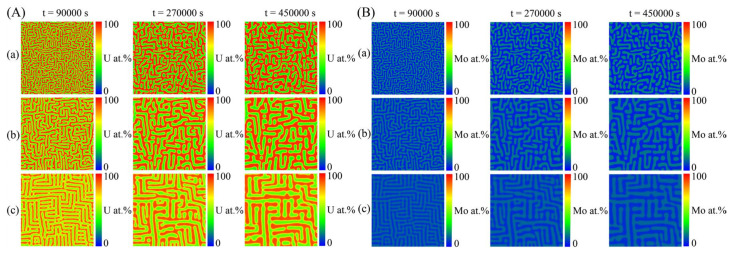
Simulation results of the spinodal decomposition in U-10 at.% Mo-20 at.% Zr alloy at different temperatures. (**A**) U-enriched γ-phase, (**B**) Mo-enriched γ-phase. Rows (**a**–**c**) correspond to the temperatures of 673 K, 773 K and 873 K, respectively.

The thermodynamic parameters describing the Gibbs free energy of the Bcc phase in the U–Mo–Zr system are listed in Table 2. Utilizing the thermodynamic database [12], the isothermal sections of the U-Mo-Zr ternary system were calculated at temperatures of 673 K, 773 K and 873 K, as depicted in Figure 5, where the red dots in the figures are the initial alloy compositions used for the following phase-field simulations. It can be seen that the U-Mo-Zr alloy has wide two-phase and three-phase miscibility gaps within the 673–873 K range. The initial composition to simulate the spinodal decomposition of the U-Mo-Zr alloy is listed in Table 3. Figure 6A illustrates the temporal evolution of the microstructure in the U-10 at.% Mo-20 at.% Zr alloy at temperatures of 673 K, 773 K and 873 K. In the figure, the U-enriched γ-phase is represented by the dark red regions. The simulated microstructures of the U-10 at.% Mo-20 at.% Zr alloy at different temperatures are shown in Figure 6B, in which the Mo-enriched γ-phase is presented in a light-yellow color. The U-enriched γ-phase in the alloy exhibits a maze-like or plane-like shape, which is irregular and interconnected with a preferential alignment along the horizontal and vertical directions during spinodal decomposition.

**Figure 7:**
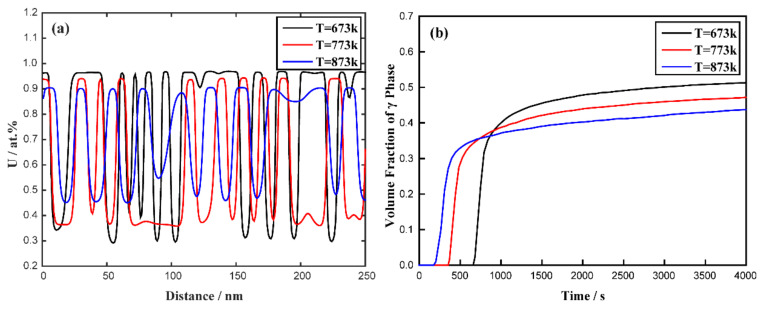
The calculated results of the U-10 at.% Mo-20 at.% Zr alloy at different temperatures (673 K, 773 K and 873 K): (**a**) the concentration profiles at the time of 4500 s; (**b**) the volume fraction as a function of time.

When the aging time is 4500 s, the evolutions of the concentration profile of the U-10 at.% Mo-20 at.% Zr alloy at different temperatures are shown in Figure 7a. With the increase in temperature, the fluctuation range of the concentration of the U-10 at.% Mo-20 at.% Zr alloy decreases, which is different from the result of the U-Mo binary system. Figure 7b illustrates the evolution of the volume fraction of the U-enriched γ-phase in the U-10 at.% Mo-20 at.% Zr alloy at temperatures of 673 K, 773 K and 873 K. It is clearly seen that the equilibrium volume fraction of the U-enriched γ-phase in the U-10 at.% Mo-20 at.% Zr alloy decreases, while the rate of phase separation increases with increasing temperature.

### 3.2. The Effect of Radiation on Spinodal Decomposition of U-Mo and U-Mo-Zr Alloys

**Figure 8:**
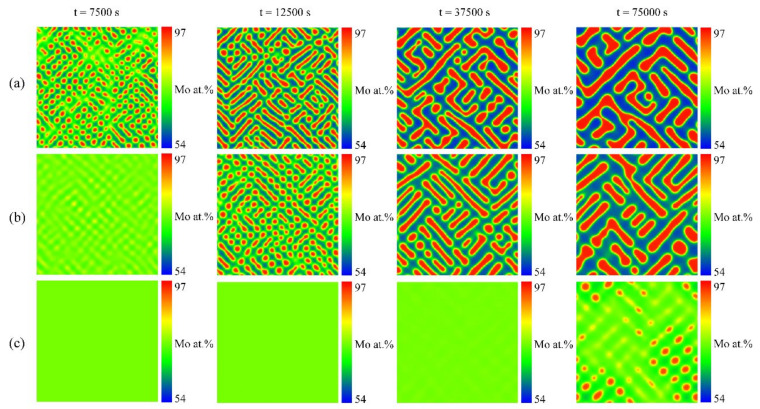
Simulation results of the spinodal decomposition in U-75 at.% Mo alloy at 1273 K under different irradiation intensities. Rows (**a**–**c**) correspond to dose rates of 5.0 × 10^−5^ dap/s, 1.0 × 10^−4^ dap/s and 2.0 × 10^−4^ dap/s, respectively.

**Figure 9:**
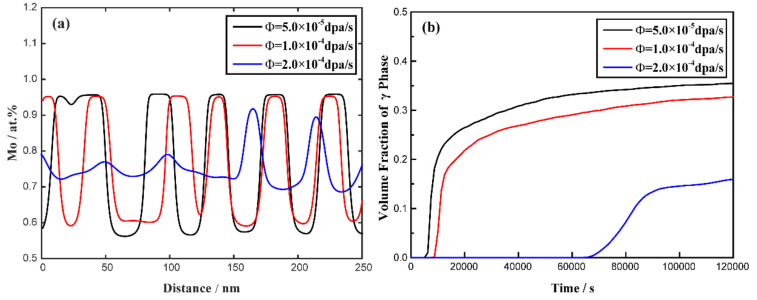
(**a**) The calculated concentration profiles at the time of 75,000 s and (**b**) volume fraction as a function of time for the U-75 at.% Mo alloy at 1273 K under different dose rates.

Figure 8 shows the microstructure evolution of the spinodal decomposition of the U-75 at.% Mo alloy at 1273 K, with dose rates of 5.0 × 10^−5^ dap/s, 1.0 × 10^−4^ dap/s and 2.0 × 10^−4^ dap/s. Compared with the simulated results without irradiation in Figure 2, it can be clearly seen that the orientation of the γ-phase does not change with the increase in irradiation intensity, while the microstructure morphology tends to change from being island-shaped to an interconnected strip shape. Cascade mixing increases the size of the γ-phase during the initial stage of spinodal decomposition. Under the effect of irradiation, a long incubation period is needed to overcome localized cascade mixing and initiate the decomposition, and the time required for spinodal decomposition increases with increasing irradiation intensity. Figure 9a illustrates the concentration evolution of the U-75 at.% Mo alloy at 1273 K under different irradiation intensities at the time of 75,000 s. It can be easily found that the Mo-lean and Mo-rich phases in the U-75 at.% Mo alloy can reach equilibrium compositions of 56 at.% and 96 at.% at 5.0 × 10^−5^ dap/s; 59 at.% and 95 at.% at 1.0 × 10^−4^ dap/s; and 68 at.% and 91 at.% at 2.0 × 10^−4^ dap/s. The evolution of the volume fraction of the Mo-enriched γ-phase in the U-75 at.% Mo alloy at a temperature of 1273 K at various dose rates is presented in Figure 9b. The volume fraction of the Mo-enriched γ-phase decreases from 35 % without irradiation to 32 % with a dose rate of 5.0 × 10^−5^ dap/s, which indicates that the spinodal decomposition is inhibited by the increase in irradiation intensity.

**Figure 10:**
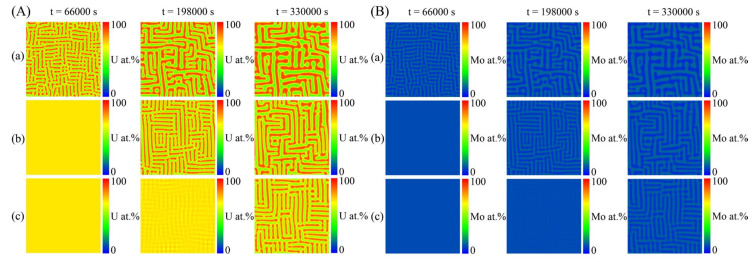
Simulation results of the spinodal decomposition in U-10 at.% Mo-20 at.% Zr alloy at 873 K under different dose rates. (**A**) U-enriched γ-phase, (**B**) Mo-enriched γ-phase. Rows (**a**–**c**) correspond to dose rates of 0.0 dap/s, 1.0 × 10^−4^ dap/s and 2.0 × 10^−4^ dap/s, respectively.

**Figure 11:**
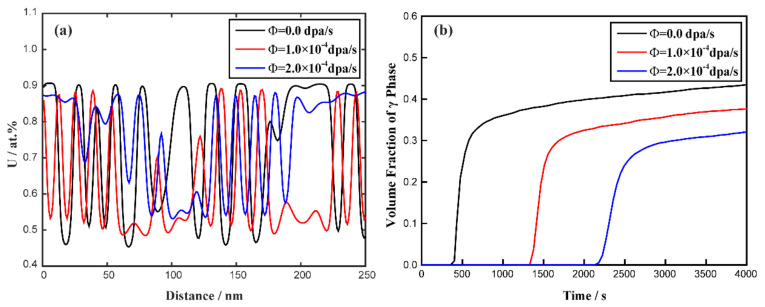
(**a**) The calculated concentration profiles at the time of 3300 s and (**b**) volume fraction as a function of time for the U-10 at.% Mo-20 at.% Zr alloy at 873 K under different dose rates.

Figure 10 shows the simulated results of the spinodal decomposition of the U-10 at.% Mo-20 at.% Zr alloy at 873 K under dose rates of 0.0 dpa/s, 1.0 × 10^−4^ dap/s and 2.0 × 10^−4^ dap/s. It can be seen from Figure 10 that the irradiation intensities have an inapparent influence on the morphology of the U-10 at.% Mo-20 at.% Zr alloy, which still shows a maze-like or plane-like pattern. As the irradiation intensity increases, the de-mixing process prevails over the local cascade mixing and enhances the orientated growth along the horizontal or vertical directions to minimize the elastic energy. Figure 11a shows the concentration evolution of the U-10 at.% Mo-20 at.% Zr alloy at different dose rates at the time of 3300 s. It can be seen that the U-lean and U-rich phase in the U-10 at.% Mo-20 at.% Zr alloy reaches the equilibrium compositions of 45 at.% and 91 at.% at 0.0 dap/s; 49 at.% and 89 at.% at 1.0 × 10^−4^ dap/s; and 53 at.% and 87 at.% at 2.0 × 10^−4^ dap/s. As the dose rates increase, the equilibrium composition range of the U-enriched γ-phase shrinks. The volume fraction evolution of the U-10 at.% Mo-20 at.% Zr alloy at different irradiation intensities is presented in Figure 11b. The figure illustrates that as the dose rate increases, a long incubation time is required for the spinodal decomposition to develop, and the volume fraction of the U-enriched γ-phase at 2.0 × 10^−4^ dap/s decreases from 43% to 32%.

**Figure 12:**
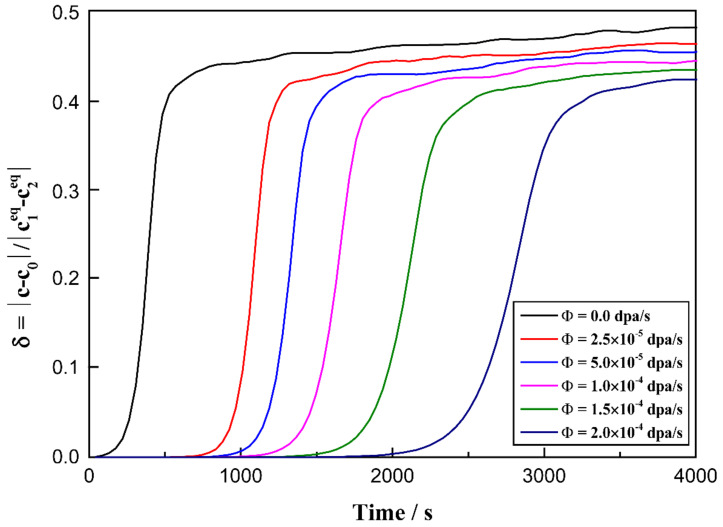
The relationship between δ and time for the U-10 at.% Mo-20 at.% Zr alloy under different irradiation conditions at 873 K.

To investigate the rate of the spinodal decomposition, the ratio of the absolute composition deviation and the miscibility gap (*δ*) can be defined as follows:

(17) $$\delta =\frac{\left|c_{t}^{Max}-c_{0}\right|}{\left|c_{1}^{eq}-c_{2}^{eq}\right|}$$

where $c_{t}^{Max}$ is the maximum instantaneous concentration at *t*, *c_0_* is the initial concentration and $c_{1}^{eq}$ and $c_{2}^{eq}$ indicate the equilibrium concentrations of the Mo-rich and Mo-lean phases. Figure 12 shows the change in the *δ* value versus time for the U-10 at.% Mo-20 at.% Zr alloy under various irradiation conditions at 873 K. According to Figure 12, one can easily find that the process of spinodal decomposition is retarded with the increase in irradiation intensity.

### 3.3. The Effect of Radiation on Phase Diagrams in U-Mo and U-Mo-Zr Alloys

**Figure 13:**
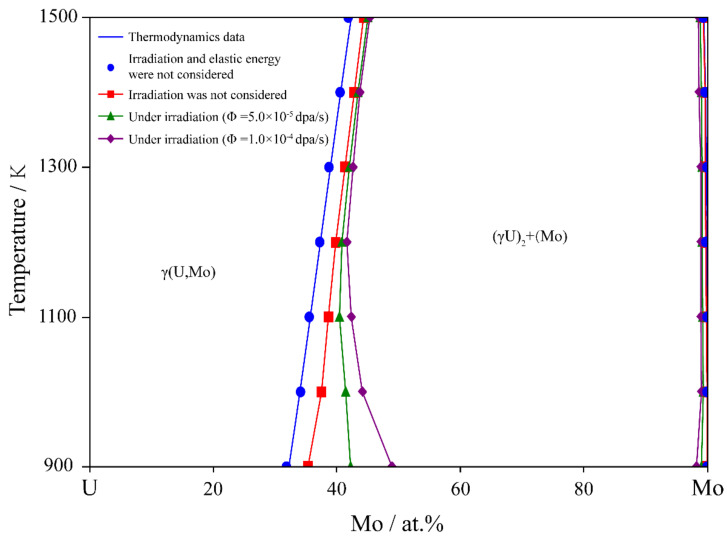
The influence of irradiation on the miscibility gap in U-Mo alloy.

Based on the phase-field simulation at different temperatures, the equilibrium compositions of the U-Mo alloy under various dose rates can be determined. Figure 13 shows the impact of irradiation on the miscibility gap of the Bcc phase in the U-Mo alloy for one-month aging under various irradiation conditions. In the absence of irradiation and elastic energy, the simulation results are consistent with the equilibrium phase diagram. The red curve is the simulation results considering the elastic energy without irradiation (usually called a coherent phase diagram), showing a slight shrinkage of the miscibility gap. The green and purple curves indicate the results considering the elastic energy at dose rates of 5.0 × 10^−5^ dap/s and 1.0 × 10^−4^ dap/s, respectively. Clearly, the range of compositions in the miscibility gap decreases as the irradiation intensity increases.

**Figure 14:**
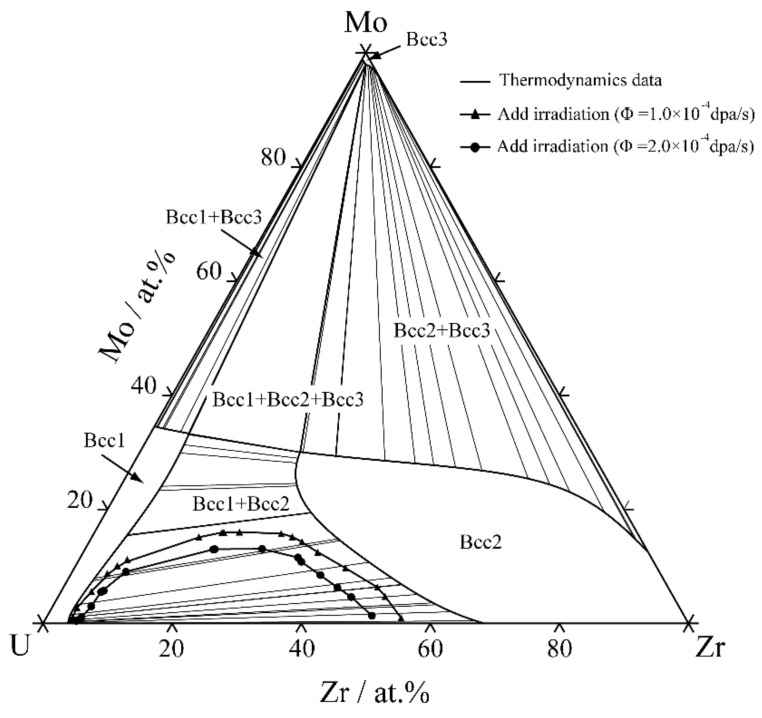
The influence of irradiation on the miscibility gap in U-Mo-Zr alloy at 873 K.

The equilibrium compositions of spinodal decomposition for ternary U-Mo-Zr alloys at various temperatures were also be calculated. The two-phase regions of the Bcc spinodal decomposition under dose rates of 1.0 × 10^−4^ dap/s and 2.0 × 10^−4^ dap/s for different conditions were merged with the equilibrium phase diagram and are shown in Figure 14. As can be seen, the two-phase regions on the U-enriched side gradually decrease with the increase in irradiation intensities, which imply that the miscibility gap in the ternary U-Mo-Zr alloy is also be reduced when subjected to irradiation, leading to the expansion of the Bcc single-phase region at the U-Mo sub-binary side.

**Figure 15:**
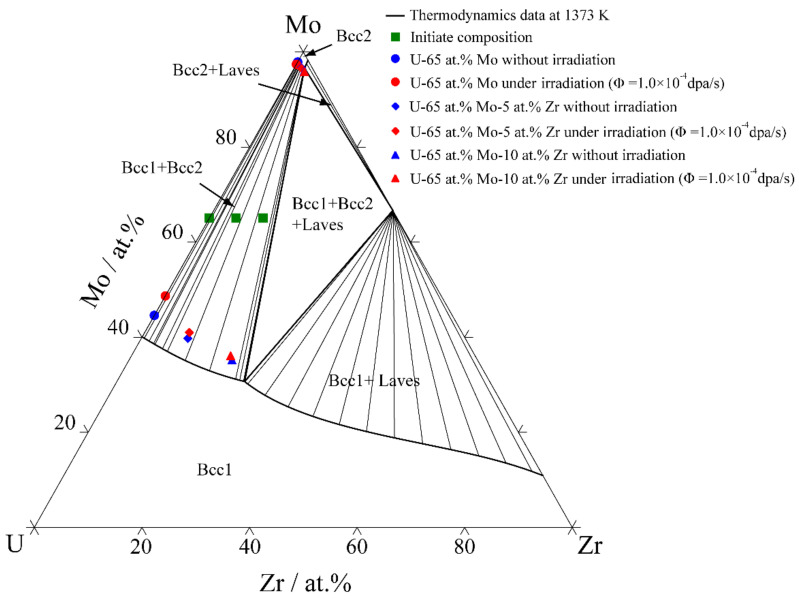
The influence of different Zr compositions on miscibility gap in U-Mo-Zr alloy under irradiation at 1373 K.

The equilibrium compositions of the U-65 at.% Mo, U-65 at.% Mo-5 at.% Zr and U-65 at.% Mo-10 at.% Zr alloys after aging for one month under different irradiation conditions were calculated. Figure 15 shows a comparison of the phase boundaries of the isothermal section in the U-Mo-Zr system at 1373 K. The figure illustrates that the changes in the simulated equilibrium compositions conform to the trend of the timeline calculated using the CALPHAD database. In the U-65 at.% Mo alloy, the range of the miscibility gap at 1.0×10^−4^ dap/s decreases from 0.5331 to 0.4874. In the U-65 at.% Mo-5 at.% Zr alloy, the width of the miscibility gap is 0.5689 without irradiation, while the width is 0.5550 when the irradiation intensity is 1.0 × 10^−4^ dap/s. In the U-65 at.% Mo-10 at.% Zr alloy, the range of the miscibility gap is 0.6076 without irradiation, while the width is 0.5926 when the irradiation intensity is 1.0 × 10^−4^ dap/s. Under the same irradiation intensity of 1.0 × 10^−4^ dap/s, the miscibility gap of U-65 at.% Mo and U-65 at.% Mo-5 at.% Zr shrinks by 8.57 % and 2.44 %, and by 2.47 % in U-65 at.% Mo-10 at.% Zr.

## 4. Discussion

This study utilizes the phase-field model, coupled with the micro-elastic theory and cascade mixing, to investigate spinodal decomposition in U-Mo and U-Mo-Zr alloys. The U-enriched γ-phase and Mo-enriched γ-phase obtained via spinodal decomposition are Bcc phases with a cube crystal structure. The lattice misfit between the phases during spinodal decomposition, leading to elastic energy, was computed. The U-Mo and U-Mo-Zr alloy systems’ elastic anisotropy explains why the isolated domains have a tendency to align in opposite orientations. The expression of the elastic modulus can be shown as follows [71]:

(18) $$E\left(\theta \right)=\left(\frac{C_{11}+C_{12}}{2}+\frac{C_{44}}{2\mu }\right)+4C_{44}\left(1-\frac{1}{\mu }\right)\sin^{2}\theta \cos^{2}\theta$$

**Figure 16:**
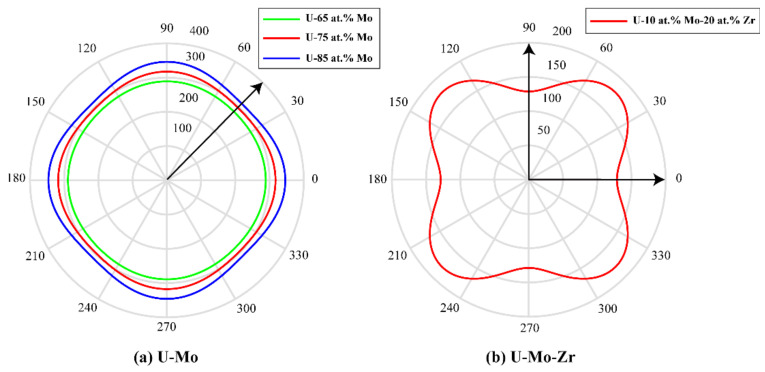
Calculated diagram of Young’s modulus: (**a**,**b**).

Here, $\mu =2C_{44}/(C_{11}-C_{12})$ is the orientation factor. As a function of the orientation, Figure 16a,b show the curves of the variation of *E* in the U-Mo and U-Mo-Zr alloys. Figure 16a shows that the Young’s modulus is lower in the <11> crystallographic orientation compared to other orientations in the U-Mo alloy. Therefore, the γ-phase of the U-Mo alloy evolves with a preferential alignment along the <11> direction, which is identical to the results of the phase-field simulation depicted in Figure 2. The coarsening of the γ-phase in U-Mo alloys can be clearly seen in the simulation results, i.e., the dissolution of small particles and coalescence, which is mainly caused by Ostwald ripening in this period [71,72]. At the early stage, the γ-phase of the U-Mo alloy preferentially aligns along the <11> direction to reduce elastic strain energy. Moreover, different compositions of Mo can lead to the various geometrical shapes of particles due to the elastic anisotropic growth bias.

The influence of temperature on the microstructure in the U-75 at.% Mo alloy (Figure 3 and Figure 4) indicated that, at higher temperatures, spinodal decomposition tends to evolve faster due to the faster thermal diffusivities, regardless of the lower chemical driving force [73]. Therefore, there exist larger particles of the Mo-enriched γ-phase at high temperatures. With the increase in the simulation time, the volume fraction of decomposed phases increases slowly and approximates the equilibrium value.

According to the diagram of Young’s modulus of the U-10 at.% Mo-20 at.% Zr alloy in Figure 16b, it is obvious that Young’s modulus has minimum values in the crystallographic directions of <10> and <01>. Therefore, the elastic energy of the γ-phase in the U-10 at.% Mo-20 at.% Zr alloy prefers to grow along the <10> and <01> directions, and finally develops an interlaced horizontal and vertical two-phase structure, which also coincides with the phase-field simulation results shown in Figure 6. With the increase in the simulation time, precipitated phase coarsening occurs through the mechanism of Ostwald ripening. As temperature rises, the amount of time needed for spinodal decomposition to achieve an equilibrium state reduces. The concentration fluctuation range and volume fraction of the U-rich phase after spinodal decomposition decrease with the increase in the simulated temperature, which are contrary to the simulation results of the U-75 at.% Mo alloy. According to the isothermal sections of the U-Mo-Zr alloy, the miscibility gap of the two-phase region of the Bcc phase in the U-rich corner decreases with increasing temperature, which has a greater impact on the equilibrium composition after spinodal decomposition. Therefore, in the U-Mo-Zr alloy, the contraction of two-phase regions leads to a decrease in the concentration fluctuation range and volume fraction with the increase in temperature.

In the simulation, the miscibility gap width in the equilibrium phase diagram can be expressed as $\Delta {\bar{c}}^{eq}=\left|{\bar{c}}_{1}^{eq}-{\bar{c}}_{2}^{eq}\right|$, in which ${\bar{c}}_{1}^{eq}$ and ${\bar{c}}_{2}^{eq}$ represent the equilibrium compositions. According to Figure 8 and Figure 9, in the U-75 at.% Mo alloy, the miscibility gap, $\Delta {\bar{c}}^{eq}$, is 0.40 at a dose rate of 5.0 × 10^−5^ dpa/s, while $\Delta {\bar{c}}^{eq}=0.23$ at a dose rate of 2.0 × 10^−4^ dpa/s. In Figure 10 and Figure 11, the range of the miscibility gap, $\Delta {\bar{c}}^{eq}$, is 0.46 without irradiation, decreasing to 0.34 at a dose rate of 2.0 × 10^−4^ dpa/s. With the increasing irradiation intensity, the difference in equilibrium compositions ($\Delta {\bar{c}}^{eq}$) between the U-Mo and U-Mo-Zr alloys during spinodal decomposition decreases significantly. According to Figure 12, it can be obviously seen that the irradiation reduces the amplitude of the composition fluctuation and the rate of the spinodal decomposition has been slowed down.

As mentioned above, increased dosage rates lead to a higher occurrence of cascade-mixing substitution events. These events compel the transfer of atoms from the matrix to precipitates, resulting in changes to the equilibrium composition and volume fraction. The influence of irradiation on the microstructure is mainly reflected in that the irradiation-induced cascade mixing effect, which will enhance the local mixing of atoms and thus affect the phase transformation process. In the absence of dose rates, spinodal decomposition induces the spontaneous breakdown of the metastable supersaturated solid solution into two-phase mixtures with an identical crystal structure, resulting in a reduction in the system’s free energy. However, under irradiation conditions, the irradiation-induced ballistic mixing effect leads to more atomic position exchange within a limited mean repositioning distance, which inhibits the diffusion of local atoms [74]. The irradiation-induced ballistic mixing results in the re-mixing of local atoms, leading to a certain degree of average composition alteration. This is contrary to the steady de-mixing mechanism or spinodal decomposition. Thus, the spinodal decomposition of U-75 at.% Mo and U-10 at.% Mo-20 at.% Zr alloys with different irradiation conditions can be assumed as the competitive outcome of irradiation-induced ballistic mixing and de-mixing [75]. The experimental results [76] showed that irradiation has the potential to decrease the spinodal decomposition degree as well as the solute composition fluctuation, and their conclusions were consistent with the above simulation results. As the irradiation dose rate increases, the more frequent cascade mixing effects lead to changes in the equilibrium composition of the γ-phase, forcing the atoms to exchange from the matrix phase to the precipitated phase and vice versa [58]. The simulation results of spinodal decomposition in the U-Mo and U-Mo-Zr alloys under irradiation agree with Schwen et al.’s [77] results of binary collision coupled with Monte Carlo simulations considering the influence of cascade mixing, whose study demonstrated that as the irradiation rates increase, the extent of phase separation diminishes. When the irradiation rates are large enough, the phase separation in the alloys is prohibited and the alloys remain mixed.

As is shown in Figure 13 and Figure 14, the range of the miscibility gaps in both U-Mo and U-Mo-Zr alloys shrinks due to the irradiation-induced cascade mixing effect. As the intensities of the irradiation increase, a narrower miscibility gap appears. The calculation results of the U-Mo alloy are consistent with the steady-state dynamical phase diagram previously reported for the U-Mo binary system under irradiation [78], indicating a decrease in the miscibility gap ((γU)1 + Mo) as dose rates increase, although the elastic energy is not considered in the calculation. It is obvious that the cascade mixing induced by irradiation is opposite to thermodynamically driven dynamic process, resulting in a reduction in the composition range of spinodal decomposition. The reason is that the irradiation has a hindering effect on the local diffusion, forcing the system to achieve a new local dynamic equilibrium, and ultimately leading to a change in the miscibility gap and the retardation of spinodal decomposition.

According to Figure 15, under irradiation, the variation in the range of the miscibility gap decreases with the increase in Zr contents, indicating that the addition of Zr could counteract the influence of irradiation. According to the thermodynamic database [12], the thermodynamic driving force of the U-65 at.% Mo-10 at.% Zr alloy is greater than that of the U-65 at.% Mo-5 at.% Zr alloy. The thermodynamic driving force of the U-65 at.% Mo-5 at.% Zr alloy is greater than that of the U-65 at.% Mo alloy. The addition of Zr could raise the driving force of spinodal decomposition, and enhance the ability of atoms to overcome local cascade mixing. In addition, irradiation can disrupt the initial equilibrium state, which increases the entropy of the γ-phase, reducing the effective free energy of the alloy [79,80]. Lu et al. [22] showed that, due to the decrease in the effective free energy of the Zr-rich phase compared to the Zr-poor phase, the volume fraction of the Zr-rich γ phase in U-Zr alloys increases with higher dose rates. The molecular dynamics simulations of Zhou et al. [81] and Moore et al. [82] for a U-10 wt.% Zr alloy shows that the phase separation of U and Zr in the U-10 wt.% Zr alloy has a large thermodynamic driving force at close to 1000 K, and the cascade mixing effect can accelerate the phase separation of the U-rich phase and Zr-rich phase. Therefore, the proper addition of Zr can effectively weaken the effect of irradiation on the spinodal decomposition of the U-Mo-Zr alloy and the shrinkage of the miscibility gap.

## 5. Conclusions

By coupling with the micro-elasticity model and cascade mixing model, the phase-field method has been used to investigate the spinodal decomposition of U-Mo and U-Mo-Zr alloys under various irradiation conditions based on the CALPHAD database. The influence of the alloys’ composition, aging temperature and irradiation intensity on the miscibility gap, microstructure and volume fraction have been investigated. The simulation results facilitate the comprehension of spinodal decomposition in U-Mo and U-Mo-Zr alloys, as well as the development of metallic fuels suitable for high-temperature and irradiation environments. The results are the following:

1. As the temperature increases, the amplitude of component fluctuations and the volume fraction of the Mo-enriched γ-phase in the U-Mo binary alloy gradually increase after spinodal decomposition, and the morphology is in interconnected plane-like shapes. In the U-Mo-Zr ternary alloy, as the temperature increases, the microstructure obtained through spinodal decomposition is in interconnected maze-like shapes. Due to the decrease in the range of the miscibility gap with increasing temperature, the amplitude of component fluctuations and the volume fraction of the U-enriched γ-phase decrease.
2. Under the effect of elastic energy, the γ-phase in the U-75 at.% Mo alloy evolves with a preferential alignment along the <11> crystallographic orientation and the morphology is in interconnected plane-like shapes, while the orientation of the microstructure in the U-10 at.% Mo-20 at.% Zr alloy grows along the <10> and <01> directions, and the morphology is in interconnected maze-like shapes. Different irradiation intensities have little effect on the microstructure and morphology of U-Mo and U-Mo-Zr alloys after spinodal decomposition. As the irradiation intensity increases, the spinodal decomposition rate of U-Mo alloys and U-Mo Zr alloys decreases; the size and volume fraction of the γ-phase decrease; and the range of the miscibility gap after spinodal decomposition also decreases.
3. Under irradiation conditions, the cascade mixing effect induced by irradiation “remixes” local atoms, suppressing their diffusion, contrary to the mechanism of spinodal decomposition. Therefore, the spinodal decomposition of U-Mo and U-Mo-Zr alloys under irradiation is the result of competition between thermodynamic spontaneous spinodal decomposition and irradiation-induced cascade mixing.

## Figures and Tables

**Table 2:** The thermodynamic parameters of the U–Mo-Zr system [12].

BCC_A2: (Mo,U,Zr)_1_(Va)_3_
$L_{Mo,U}^{0,\mathrm{bcc}}=11874+3.571*T$
$L_{Mo,U}^{1,\mathrm{bcc}}=-5018+19.089*T$
$L_{Mo,U}^{2,\mathrm{bcc}}=7000-2.534*T$
$L_{U,Zr}^{0,\mathrm{bcc}}=51430.744-40.504*T$
$L_{U,Zr}^{1,\mathrm{bcc}}=6697.702$
$L_{U,Zr}^{2,\mathrm{bcc}}=1482.94$
$L_{Mo,Zr}^{0,\mathrm{bcc}}=17935.985+3.102*T$
$L_{Mo,Zr}^{1,\mathrm{bcc}}=-990.991+4.299*T$
$L_{Mo,U,Zr}^{0,\mathrm{bcc}}=50000$
$L_{Mo,U,Zr}^{1,\mathrm{bcc}}=-70000+30*T$
$L_{Mo,U,Zr}^{2,\mathrm{bcc}}=-310000+120*T+2*T*LN(T)$

**Table 3:** Samples of alloys under different initial conditions.

Alloy Composition	Aging Temperature	Irradiation Condition
U-65 at.% Mo	1273 K	0 dap/s
U-75 at.% Mo	923 K	0 dap/s
U-75 at.% Mo	1273 K	0 dap/s
U-75 at.% Mo	1473 K	0 dap/s
U-85 at.% Mo	1273 K	0 dap/s
U-65 at.% Mo	1373 K	1.0 × 10^−4^ dap/s
U-75 at.% Mo	1273 K	5.0 × 10^−5^ dap/s
U-75 at.% Mo	1273 K	1.0 × 10^−4^ dap/s
U-75 at.% Mo	1273 K	2.0 × 10^−4^ dap/s
U-10 at.% Mo-20 at.% Zr	673 K	0 dap/s
U-10 at.% Mo-20 at.% Zr	773 K	0 dap/s
U-10 at.% Mo-20 at.% Zr	873 K	0 dap/s
U-10 at.% Mo-20 at.% Zr	873 K	1.0 × 10^−4^ dap/s
U-10 at.% Mo-20 at.% Zr	873 K	2.0 × 10^−4^ dap/s
U-65 at.% Mo-5 at.% Zr	1373 K	1.0 × 10^−4^ dap/s
U-65 at.% Mo-10 at.% Zr	1373 K	1.0 × 10^−4^ dap/s

## Data Availability

Data are contained within the article.
